# No trait anxiety influences on early and late differential neuronal responses to aversively conditioned faces across three different tasks

**DOI:** 10.3758/s13415-022-00998-x

**Published:** 2022-03-29

**Authors:** Sebastian Schindler, Jana Heinemann, Maximilian Bruchmann, Robert Moeck, Thomas Straube

**Affiliations:** 1 Institute of Medical Psychology and Systems Neuroscience, University of Muenster and University Hospital Münster, Von-Esmarch-Str. 52, 48149 Münster, Germany; 2 Otto Creutzfeldt Center for Cognitive and Behavioral Neuroscience, University of Muenster, Münster, Germany

**Keywords:** Fear conditioning, Trait anxiety, EEG/ERP, P1, N170, EPN, LPP

## Abstract

The human brain's ability to quickly detect dangerous stimuli is crucial in selecting appropriate responses to possible threats. Trait anxiety has been suggested to moderate these processes on certain processing stages. To dissociate such different information-processing stages, research using classical conditioning has begun to examine event-related potentials (ERPs) in response to fear-conditioned (CS +) faces. However, the impact of trait anxiety on ERPs to fear-conditioned faces depending on specific task conditions is unknown. In this preregistered study, we measured ERPs to faces paired with aversive loud screams (CS +) or neutral sounds (CS −) in a large sample (*N* = 80) under three different task conditions. Participants had to discriminate face-irrelevant perceptual information, the gender of the faces, or the CS category. Results showed larger amplitudes in response to aversively conditioned faces for all examined ERPs, whereas interactions with the attended feature occurred for the P1 and the early posterior negativity (EPN). For the P1, larger CS + effects were observed during the perceptual distraction task, while the EPN was increased for CS + faces when deciding about the CS association. Remarkably, we found no significant correlations between ERPs and trait anxiety. Thus, fear-conditioning potentiates all ERP amplitudes, some processing stages being further modulated by the task. However, the finding that these ERP differences were not affected by individual differences in trait anxiety does not support theoretical accounts assuming increased threat processing or reduced threat discrimination depending on trait anxiety.

**Supplementary Information:**

The online version contains supplementary material available at 10.3758/s13415-022-00998-x.

## Introduction

Faces are salient social stimuli that exhibit unique identity information. The detection of face identities that signal threat or danger is an important skill to trigger an appropriate response and to avoid damage. A human face’s complex set of features may require attentional selection of those features required for a specific task: for example, judging whether a face is familiar, young or old, male or female, or displays affective information. Even inherently neutral faces can acquire affective information (e.g., associating faces with a loud scream), whereby learning of these associations and emotional responses are influenced by the situational context and individual differences in trait anxiety (Lonsdorf & Merz, 2017). Trait anxiety characterizes a disposition to respond with concerns, troubles, and worries to various situations (Bishop, 2008; Spielberger, 1972; Spielberger et al., 1999). Trait anxiety has been suggested to be associated with increased attention to potential threat cues (Fox et al., 2002; MacLeod & Clarke, 2015; Mathews & Mackintosh, 1998; Mathews & MacLeod, 2002; Yiend & Mathews, 2001). Specifically, high trait-anxious people are reasoned to exhibit a hypersensitive threat-detection system (Bar-Haim et al., 2007; Bishop, 2008), leading to specific processing biases (Bishop, 2009; Pacheco-Unguetti et al., 2010; Wieser & Keil, 2020). However, other studies suggest that anxious individuals may exhibit more attentional suppression of conditioned threats (Kappenman et al., 2021) or reduced differentiation of threatening and neutral stimuli (Dunsmoor & Paz, 2015; Stegmann et al., 2020). Furthermore, individual differences in trait anxiety are reasoned to be associated with fear-conditioning mechanisms explaining an early phasic response to threat and a later lack of regulation (Indovina et al., 2011). Thus, studies are only partially consistent, and it is an ongoing question how trait anxiety affects threat processing, such as fear-conditioned faces.

Event-related potentials (ERPs) are well suited to study at which stage of the visual processing hierarchy, trait anxiety biases processing of threat-related faces. Early and late ERP components are markers of distinct face and emotion processing stages. First, the occipital P1 reflects early perceptual processing and differentiation of visual stimuli (Hopfinger & Mangun, 1998; Luck & Hillyard, 1994; Vogel & Luck, 2000). The face-sensitive N170 component represents an early stage of facial structure encoding with enlarged amplitudes for faces compared with objects (Eimer, 2011). The following early posterior negativity (EPN) is characterized by early attentional selection and indicates increased processing of emotionally salient stimuli with particular sensitivity to threatening expressions (Schupp, Junghöfer, et al., 2004; Schupp, Öhman, et al., 2004; Wieser et al., 2010). Finally, the late positive potential (LPP) is an important component associated with emotion processing and elaborative and controlled processes concerning stimulus evaluation and sustained attention (Hajcak et al., 2009; Schupp et al., 2006). Trait anxiety has been found to modulate ERP components to threatening facial expressions, even though with conflicting findings of either early (P1, N170) increased or mid-latency (EPN) reduced differentiation were found concerning ERP effects (Bar-Haim et al., 2005; Holmes et al., 2008; Steinweg et al., 2021; Walentowska & Wronka, 2012; Williams et al., 2007). For neutral faces that acquired negative valence by instruction, a recent study showed no relationship between individual trait anxiety and ERP differences (Krasowski et al., 2021).

Several studies show that fear-conditioning of inherently neutral faces modulate distinct ERP components (Bacigalupo & Luck, 2018; Rehbein et al., 2014; Sperl et al., 2021; Steinberg et al., 2011, 2013). Regarding the P1 component, some findings exhibit larger amplitudes for CS + stimuli in general (Liu, Huang, et al., 2012; Liu, Keil, et al., 2012; Pizzagalli et al., 2003). This amplitude increase also has been shown for CS + faces (Muench et al., 2016; but see Sperl et al., 2021; Seligowski et al., 2018). Furthermore, increased N170 (Camfield et al., 2016; Sperl et al., 2021) and EPN (Ferreira de Sá et al., 2019) responses have been observed for fear-conditioned CS + faces. Most systematically, the LPP component has been examined in fear-conditioning studies, reporting reliably larger LPP amplitudes for fear-conditioned faces (Bacigalupo & Luck, 2018; Ferreira de Sá et al., 2019; Panitz et al., 2018; Sperl et al., 2021; Stolz et al., 2019; Wiemer et al., 2021).

Only two studies have investigated the effects of trait anxiety on electrophysiological responses to fear-conditioned facial stimuli (Panitz et al., 2018; Rehbein et al., 2015), described below. Besides these studies with faces as CS, only three additional studies investigated trait anxiety effects on ERPs to fear-conditioned stimuli. You et al. (2021) investigated ERPs to fear-conditioned Gabor patches. Larger P1 amplitudes were observed in high-trait anxious individuals for luminance-conditioned Gabor patches, suggesting that trait anxiety is specifically associated with increased P1 responses to visual features stimulating the subcortical magnocellular pathway (You et al., 2021). Another study showed that conditioned simple visual stimuli elicit increased ERP responses but found these effects unrelated to trait anxiety differences (Nelson et al., 2015). Furthermore, trait anxiety effects on ERPs during fear conditioning were examined by lateralized ERPs to aversively conditioned pictorial stimuli (Kappenman et al., 2021). The PD component increased in high trait anxious participants for conditioned threat cues, which was reasoned to index increased threat-suppression processes with increased trait anxiety (Kappenman et al., 2021). Concerning faces, a MultiCS conditioning study showed increased early MEG responses (M1 and M170) for CS + faces for high compared with low-anxious individuals (Rehbein et al., 2015). Another study found no relation between trait anxiety or fearfulness and LPP responses to CS + CS + faces, whereas trait fearfulness was correlated with fear bradycardia (Panitz et al., 2018).

Thus, the few findings regarding ERPs to fear-conditioned stimuli and trait anxiety are widely inconsistent with studies showing no effects (Nelson et al., 2015; Panitz et al., 2018) or increased amplitudes of the P1/M1 (Rehbein et al., 2015; You et al., 2021), M170 (Rehbein et al., 2015), or P_D_ components (Kappenman et al., 2021). No studies have investigated the whole sequence of different components of the ERP to the CS + , and to our knowledge, no electrophysiological study has yet examined whether associations between trait anxiety and ERP effects during fear conditioning are affected by different task demands. Effects of individual differences in the processing of threatening stimuli might only be observed under specific task conditions (Lin et al., 2021; Straube et al., 2011). Studies observing relationships of trait anxiety and early ERPs often relied on implicit emotion processing tasks (Bar-Haim et al., 2005; Holmes et al., 2008; Steinweg et al., 2021; Walentowska & Wronka, 2012). The manipulation of the level of attentional engagement with emotional faces would allow investigating whether trait anxiety effects on ERPs depend on the selection of face-related features in general, or specifically, on the selection of emotion-related features.

The current preregistered study investigated the impact of trait anxiety on early (P1, N170), mid-latency (EPN), and late (LPP) components of the ERP to aversively conditioned faces during different tasks. The experimental tasks aimed to vary the required processing mode of stimuli. The three different tasks demand increasing attention to the face stimulus and the affective information associated with the face. Participants had to respond to overlaid line orientation (without any need to attend to faces), the face gender (without any need to attend to CS status of faces), or the emotional relevance of faces (CS task). We systematically explored correlations between trait anxiety and all differential ERP modulations across the tasks. Concerning secondary ERP task effects, we registered that CS + faces should elicit task-dependent effects for the P1, EPN, and LPP but task-independent increases of the N170 component. The detailed preregistration can be retrieved in the Open Science Framework (https://osf.io/v98fk) and all raw data and paradigm information in the attached OSF project (https://osf.io/hg2w9).

## Methods

We report how we determined our sample size, all data exclusions, all inclusion/exclusion criteria, whether inclusion/exclusion criteria were established prior to data analysis, all manipulations, and all measures in the study. All deviations from the preregistered protocol are mentioned in the respective sections.

***Participants.*** In total, the data sampling plan was designed to examine 80 usable datasets, based on recommendations for neuroscience studies when relating individual differences to brain responses (Mar et al., 2013). Furthermore, concerning ERP modulations, power calculations using G*Power 3.1.7 (Faul et al., 2009) showed that sampling 80 participants exhibit a power of > 99% to detect medium effects sizes (within-subject design and α = 0.05). In total, we examined 86 participants. Six were excluded: two participants due to excessive noise in their EEG data; one due to a previous anxiety disorder; one due to a paradigm error; and two because of no clear right-handedness. All participants gave written, informed consent and received 10 euros per hour for participation. The remaining 80 participants (19 males) were on average 23.36 years (*SD* = 3.16). All participants had normal or corrected-to-normal vision and were right-handed with no reported history of neurological or psychiatric disorders. Self-reported trait anxiety ratings of the State and Trait Inventory (Spielberger et al., 1999) varied between 23 and 66 (mean = 36.46, SD = 8.17; Quartiles 31, 35, 41), comparable to similar recent studies (Kappenman et al., 2021). Please note that our previous study reported task ERP effects for the subset of the first 40 participants (Bruchmann et al., 2021).

**Fig. 1:**
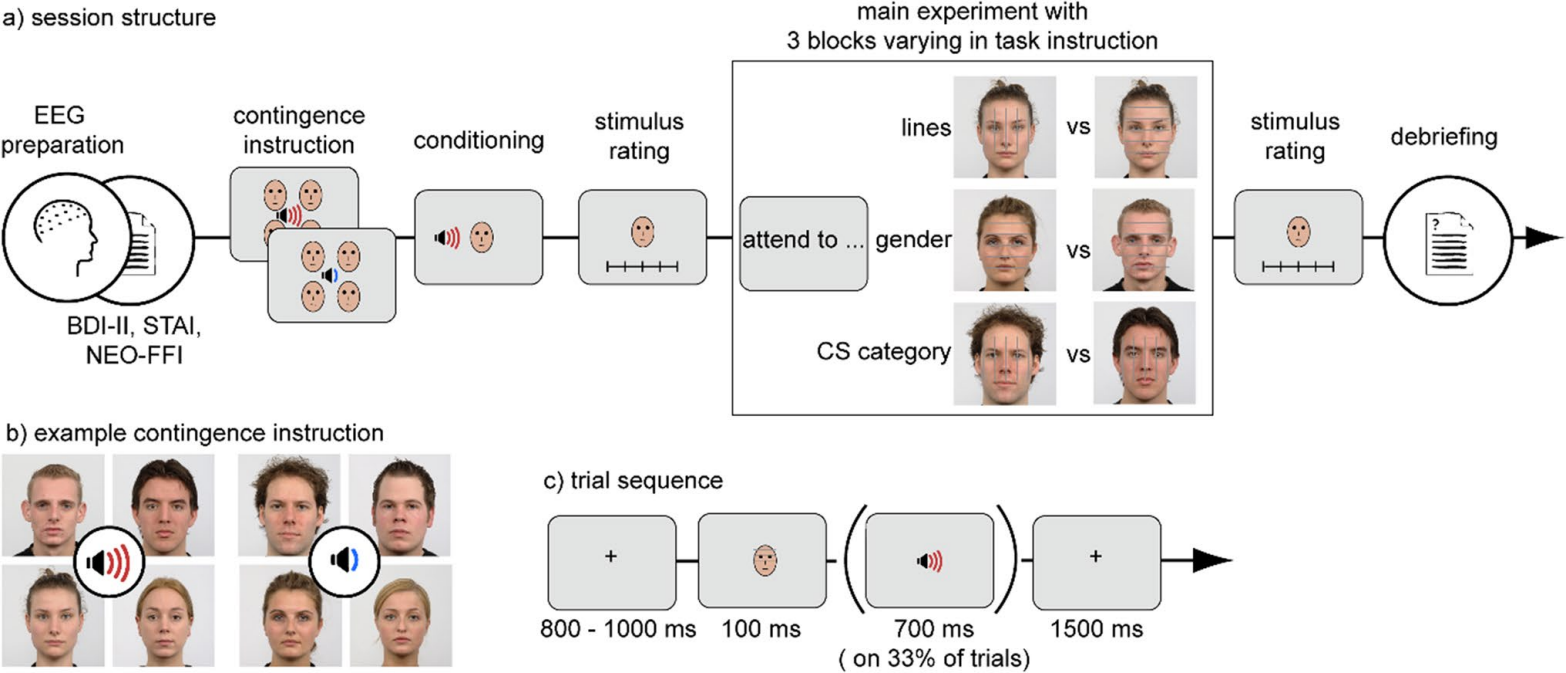
**Experiment overview**. **a)** Schematic structure of an experimental session with stimulus examples. Note that task order was counterbalanced. **b)** Example for an instructed group contingence and **c)** trial structure. Red and blue sound waves symbolize aversive screams or neutral sounds, respectively

***Stimuli.*** The facial stimuli were taken from the Radboud Faces database (Langner et al., 2010). For these stimuli, the position of the eyes and head orientation are well standardized (Langner et al., 2010). The faces were presented with a size of approximately 7.7 degrees of visual angle (bizygomatic diameter). We used coloured close-up faces. They consisted of eight identities (4 males and 4 females) with neutral expressions. Faces always were displayed with an overlay of five horizontal or vertical thin lines, evenly spaced across an area of 7.7 × 7.7 deg, which was centered on the faces’ nasion (which itself was the center of the face bitmap; Fig. 1). The lines had a thickness of 1 pixel (i.e., approximately 0.265 deg). We used auditory US stimuli, which were reported to be more effective compared with electric shocks (Sperl et al., 2016). For the aversively associated faces (CS +), we used four aversive scream sounds with 100 dB SPL, presented directly with the offset of the CS + face for 700 ms. For neutral pairings, four nonaversive sounds with 40 dB SPL were presented immediately with face offset for 700 ms. These sound pairings were presented on average in 33% of the trials, which were not used for data analyses.

***Procedure.*** Participants were instructed to avoid eye-movements and blinks during the stimulus presentation. They were prepared for the EEG, while they responded to a demographic questionnaire, as well as the BDI-II and STAI Trait questionnaire (Hautzinger et al., 2009; Spielberger et al., 1999), as well as a short version of the NEO-FFI (Körner et al., 2008). To facilitate learning, participants were instructed about the contingencies and were first exposed to the four faces paired with the CS + and the four faces paired with the CS − . Each face was presented 12 times in this conditioning block and reinforced according to a 33% schedule. Faces were presented for 100 ms, and the CS appeared directly after face offset with no overlap of the US and CS. Then, all faces were shown individually and rated according to valence and arousal, as well as asking participants to decide whether a face was paired with a loud scream or nonaversive sound. Participants were required to respond to each of the three tasks by pressing either the “x” or the “m” button, while the task order and response buttons were counterbalanced across participants. In particular, participants had to discriminate between overlaid line orientations (perceptual task), discriminate whether the face was female or male (gender task), or discriminate whether a face was paired with the loud scream or paired with the neutral sound (CS task). The exact task instructions are described in Supplementary Sect. 3. In each of the tasks, participants were first reminded about the contingencies. In all three tasks, trial structure and presentation was the same. Each trial started with a fixation cross for 800 to 1,000 ms, after which a face was presented for 100 ms. Afterwards, another fixation cross was presented for 1,500 ms where responses were recorded. Each face was repeated 24 times within one condition. In total, there were 96 trials for neutral CS − and 96 trials with neutral CS + faces, summing up to a total of 576 trials. On 33% of the trials, the face was paired with the aversive screams or with neutral sounds. These trials were discarded from further analysis. Afterward the three experimental runs, again all faces were shown and rated according to valence, arousal, and threat on a scale from 1 to 7 (1 = low, 4 = neutral, 7 = high positive valence, high arousal, or high perceived threat), as well as requesting participants to decide if a face was paired with a loud scream or nonaversive sound.

### EEG recording and preprocessing

EEG signals were recorded from 64 BioSemi active electrodes using Biosemis Actiview software (www.biosemi.com). Four additional electrodes measured horizontal and vertical eye-movement. The recording sampling rate was 512 Hz. Offline data were re-referenced to average reference, and filtered with a high-pass forward filter of 0.01 (6 db/oct) as well as a 40-Hz low-pass zero-phase filter (24 db/oct). Recorded eye movements were corrected using BESA's automatic eye-artifact correction method (Ille et al., 2002). The remaining artifacts were rejected semi-automatically by an expert rater based on an BESA’s artefact scan using the absolute threshold (120 µV), gradient (75), and low signal change (0.01). Noisy EEG sensors were interpolated using a spline interpolation procedure. The stimuli on the LCD display were found to have a trigger delay of 29 ms, which was measured by a photodiode. This delay was corrected during epoching. Filtered data were segmented from 200 ms before stimulus onset until 1,000 ms after stimulus presentation. For baseline-correction, we used the 200-ms interval before stimulus onset. On average, 2.65 (4%) electrodes were interpolated and 50 trials per condition (79%) were kept, with no differences between conditions (*Fs* < 1.64, *ps* > 0.204).

### Data analyses

Our main study goal was to test the relationship of ERP differences and individual trait anxiety scores. To this end trait, scores were correlated with the obtained differences between CS + and CS − faces using JASP (www.jasp.org). We calculated both Bonferroni-corrected inferential (adjusted *p*-value for 12 correlations < 0.004) and Bayesian Pearson correlation coefficients. For Bayesian analyses, the null hypothesis was specified as a point-null prior (i.e., standardized effect size *δ* = 0) and defined the alternative hypothesis as a Jeffreys-Zellner-Siow (*JZS*) prior, i.e., a folded Cauchy distribution centered around *δ* = 0 with the scaling factor *r* = 0.707. This scaling factor assumes a roughly normal distribution. To assign verbal labels to the strength of evidence, we followed the taxonomy suggested by Jeffreys (1961), labeling Bayes Factors with a BF_10_ of 1 as no evidence, BF_10_ between 1–3 as anecdotal evidence, 3–10 as moderate evidence, 10–30 as strong evidence, 30–100 as very strong evidence, and larger BFs as extreme evidence in favor of the alternative hypothesis. Furthermore, we added registered explorative analyses and discussion concerning the personality traits neuroticism and agreeableness in the Supplementary Materials linked in the OSF project (https://osf.io/hg2w9).

Secondary analyses were performed to validate expected behavioral and EEG scalp data effects. First, we tested differences in rated valence, arousal, and threat for negative and neutral associated faces as a manipulation check. For reaction time, hits, and ERP data, we performed two (conditioning: CS + face, CS − face) by three (task: perceptual, gender, CS + task) repeated measure ANOVAs. For post-hoc comparisons, we used Fischer’s least significant difference tests. Partial eta-squared (partial η^2^) was used to describe effect sizes (Cohen, 1988). We registered to validate our expected ERP windows for the P1 and N170 by collapsing ERPs across all conditions (Luck & Gaspelin, 2017). ERPs across all three tasks were collapsed to identify emotion effects for the EPN and LPP, typically scored as differences between emotional and neutral stimuli. Time windows were segmented from 80 to 100 ms for the P1, 120 to 170 ms for the N170, from 250 to 350 ms to investigate EPN effects, and from 400 to 700 ms to investigate LPP effects. We used two symmetrical occipital clusters for the P1 (left P9, P7, PO7, P5; right P10, P8, PO8, P6), N170, and EPN time window (left P9, P7, PO7, O1; right P10, P8, PO8, O2). The LPP component was measured over a centroparietal cluster (CP3, CP1, CPz, CP2, CP4, P3, P1, Pz, P2, P4, PO3, POz, PO4). By doing so, we deviated in time (registered N170: 120 to 170 ms; EPN 200 to 350 ms; LPP 400 to 600 ms) and space (registered P1, N170, and EPN: P9, P7, PO7, P10, P8, PO8; LPP: C1, Cz, C2, CP1, CPz, CP2; for details see Supplementary Sect. 2). Regarding behavioral data, no responses were recorded in the perceptual task for one participant, and thus this participant was excluded from behavioral data analyses. For six participants, responses were recoded since participants mistook the buttons (5 times in the emotion task, 1 time in the gender task). Exploratorily, for a manipulation check of autonomic responses to the fear-conditioning procedure, we additionally analyzed pupil dilation, which represents an established procedure to measure changes in sympathetic arousal (Bradley et al., 2008) and conditioning success (Finke et al., 2021; Korn et al., 2017), in an interval between 500 and 2,000 ms after face onset across the three tasks in 73 participants with complete eye-tracking data.

## Results

### Manipulation check

**Fig. 2:**
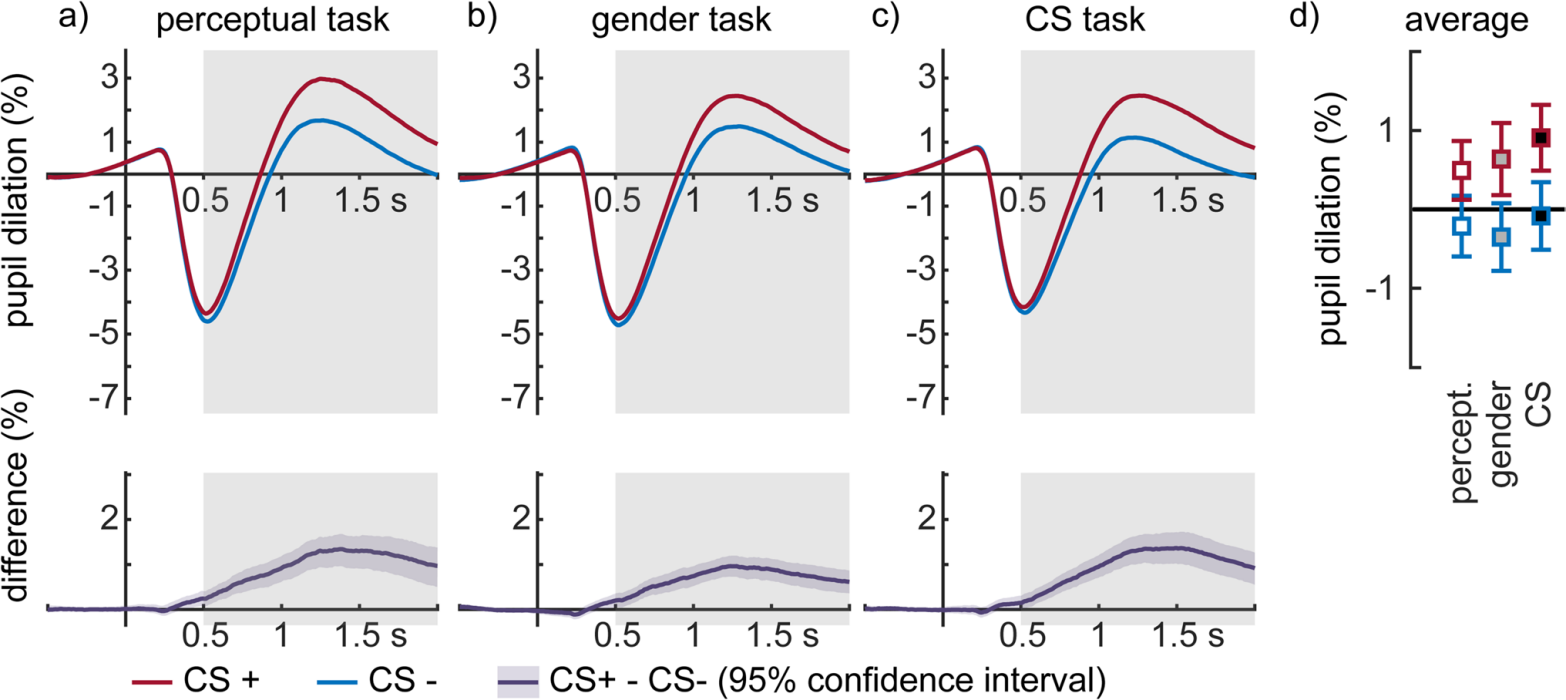
**Pupil dilation effects during the three tasks. a-c)** Differences between CS + and CS − faces. Waveforms below show difference waves with 95% confidence intervals highlighted. **d)** Mean dilation for all conditions, error bars show 95% confidence intervals

After the experiment, all faces were rated according to valence, arousal and threat, as well as requesting participants to classify faces (see procedures above). Classification accuracy was almost perfect (*M* = 0.99, *SD* = 0.03). CS + faces were rated to be significantly more negative (*M*_negative_ = 2.98, *SD* = 0.97, *M*_neutral_ = 4.54, *SD* = 1.01; *t*_(79)_ =  − 8.76, *p* < 0.001), arousing (*M*_negative_ = 4.14, *SD* = 1.37, *M*_neutral_ = 2.34, *SD* = 0.97; *t*_(79)_ = 10.62, *p* < 0.001), and threatening (*M*_negative_ = 4.07, *SD* = 1.50, *M*_neutral_ = 2.15, *SD* = 1.02; *t*_(79)_ = 10.40, *p* < 0.001) than neutral faces, while these differences were not affected by self-reported trait anxiety scores (Pearson’s *rs* < 0.100, *ps* > 0.376). Furthermore, concerning exploratory analyses of pupil dilation, there was a main effect of conditioning (*F*_(1,72)_ = 83.01, *p* < 0.001, η_P_^2^ = 0.536; Fig. 2), with larger dilation change for CS + compared with CS − faces—a main effect of task (*F*_(2,144)_ = 3.20, *p* = 0.043, η_P_^2^ = 0.043), with higher dilation during the CS task compared with the two other tasks (*ps* < 0.05), but no interaction between conditioning and the task (*F*_(2,144)_ = 2.24, *p* = 0.110, η_P_^2^ = 0.030). Pupil dilation differences were not correlated with trait anxiety in any task (Pearson's *rs* < 0.113, *ps* > 0.341).

### Experimental tasks, behavioral results

Regarding hit rate, the number of correct choices was not affected by conditioning (*F*_(1,78)_ = 2.17, *p* = 0.145, η_P_^2^ = 0.027), but a significant effect of task was found (*F*_(1.53,119.11)_ = 7.04, *p* = 0.003, η_P_^2^ = 0.083), and no interaction between conditioning and task (*F*_(1.22,94.83)_ = 0.50, *p* = 0.516, η_P_^2^ = 0.006). Participants had a higher accuracy in the gender compared with the CS task (*p* = 0.003) and to the perceptual task (*p* = 0.002). Regarding reaction time, a main effect of conditioning was found (*F*_(1,78)_ = 6.23, *p* = 0.015, η_P_^2^ = 0.074), and a main task effect was identified (*F*_(2,156)_ = 81.82, *p* < 0.001, η_P_^2^ = 0.512). Shorter reaction times were observed for CS + compared to CS − faces, and reaction times were significantly shorter in gender task compared with the perceptual and the CS tasks (*ps* < 0.001), and for the perceptual compared with the emotion task (*p* < 0.001). In addition, a significant conditioning x task interaction effect was found (*F*_(1.39,108.21)_ = 12.05, *p* < 0.001, partial η^2^ = 0.134). Post-hoc test showed no conditioning differences in the perceptual task (*p* = 0.053), but slower responses to CS + faces in the gender task (*p* = 0.017), and faster responses to these CS + faces in the CS task (*p* = 0.001; Table 1).

**Table 1:** Behavioral results across the three tasks

	Perceptual task		Gender task		CS task	
	CS + faces	CS − faces	CS + faces	CS − faces	CS + faces	CS − faces
Accuracy (%) (SD)	0.93 (0.05)	0.93 (0.04)	0.95 (0.07)	0.96 (0.06)	0.91 (0.13)	0.92 (0.09)
Reaction time (ms) (SD)	599 (86)	603 (88)	579 (88)	571 (88)	653 (98)	675 (89)

Notes. Reaction times were rounded to milliseconds. Standard deviations appear in parentheses below means.

### ERP results

#### P1

**Fig. 3:**
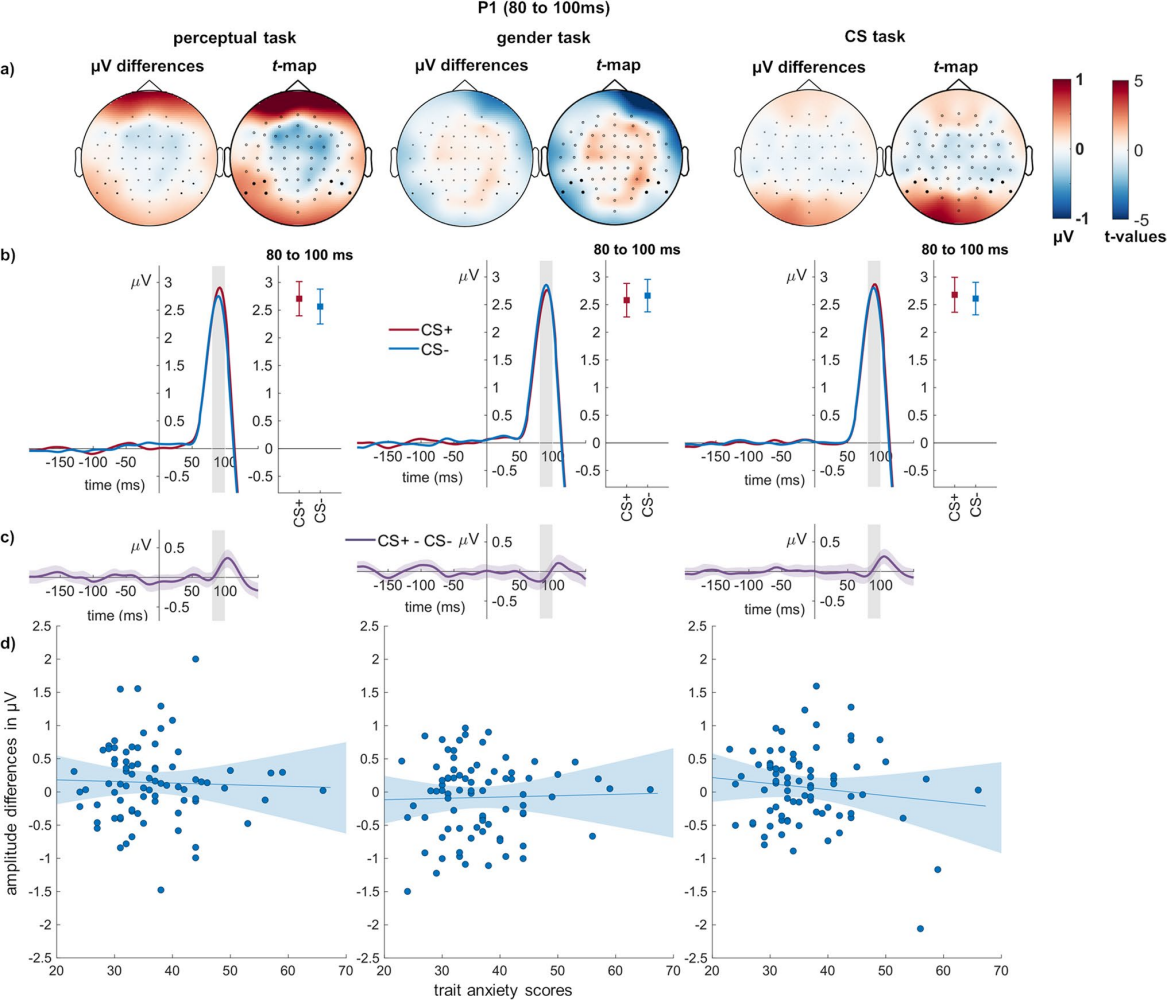
**P1 effects of conditioning and relation to individual differences. a)** In each task, scalp topographies depict the amplitude differences between CS + and CS − faces, and *t*-maps of these differences are shown. **b)** ERP waveforms show the time course over highlighted sensors. Error bars show 95% confidence intervals. **c)** Difference plots contain 95% bootstrap confidence intervals of intra-individual differences. **d)** Scatter plots of P1 differences with trait anxiety scores. The regression line and 95% confidence intervals are highlighted

With respect to the P1 component, a main effect of conditioning (*F*_(1,79)_ = 3.95, *p* = 0.050, η_P_^2^ = 0.048) but no main effect of task were observed (*F*_(2,158)_ = 0.09, *p* = 0.911, η_P_^2^ = 0.001). CS + faces elicited a larger P1 than neutral ones. There was an interaction of conditioning and task (*F*_(2,158)_ = 3.21, *p* = 0.043, η_P_^2^ = 0.039; Fig. 3). Post-hoc tests showed in the perceptual task a larger P1 for CS + compared with CS − faces (*t*_(1,79)_ = 2.68, *p* = 0.009), but no significant differences in the gender task (*t*_(1,79)_ = 0.78, *p* = 0.435) or in the CS task (*t*_(1,79)_ = 1.52, *p* = 0.132). Importantly, we tested for relationships with trait anxiety, neuroticism and agreeableness (Table 2). For the P1, all correlations with trait anxiety failed the Bonfferoni corrected threshold, and there was in all conditions at least moderate evidence against a relationship (BFs_01_ > 3).

**Table 2:** Correlation between ERP differences with individual trait anxiety scores

ERP	Correlation	Perceptual task	Gender task	CS task
P1	Pearson’s *r*	− 0.032	0.008	− 0.130
	*p* value^a^	0.775	0.940	0.250
	BF_01_	6.89	7.14	3.73
N170	Pearson's *r*	− 0.056	− 0.259	− 0.052
	*p* value^a^	0.620	0.020	0.648
	BF_01_	6.32	0.51	6.45
EPN	Pearson's *r*	− 0.181	− 0.073	0.013
	*p* value^a^	0.108	0.522	0.908
	BF_01_	2.01	5.85	7.09
LPP	Pearson’s *r*	0.177	0.138	− 0.006
	*p* value^a^	0.115	0.222	0.958
	BF_01_	2.11	3.43	7.14

Note: ^a^ Bonferroni-corrected significance threshold *p* < 0.004. BF_10_ indicates evidence in favor of the alternative hypothesis (H1) and conversely BF_01_ indicates evidence in favor of the null hypothesis of no relationship.

#### N170

**Fig. 4:**
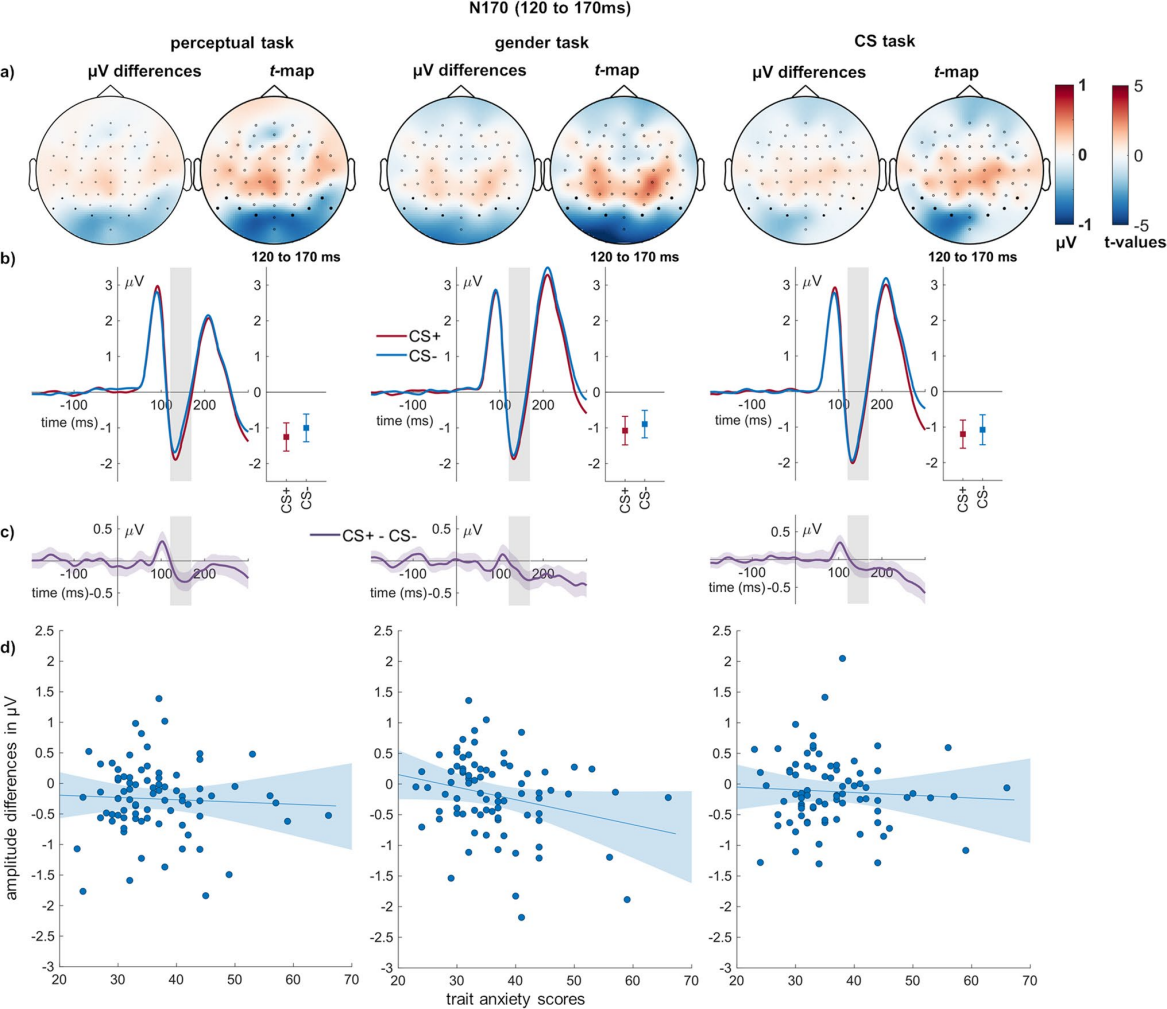
**N170 effects of conditioning and relation to individual differences. a)** In each task, scalp topographies depict the amplitude differences between CS + and CS − faces, and *t*-maps of these differences are shown. **b)** ERP waveforms show the time course over highlighted sensors. Error bars show 95% confidence intervals. **c)** Difference plots contain 95% bootstrap confidence intervals of intra-individual differences. **d)** Scatter plots of N170 differences with trait anxiety scores. The regression line and 95% confidence intervals are highlighted

Regarding the N170, there was a main effect of conditioning (*F*_(1,79)_ = 17.15, *p* < 0.001, η_P_^2^ = 0.178; Fig. 4) but no significant main effect of task (*F*_(1.83,144.17)_ = 3.13, *p* = 0.051, η_P_^2^ = 0.038). CS + faces elicited a larger N170 than CS − faces. There was no interaction of conditioning and task (*F*_(2,158)_ = 0.83, *p* = 0.439, η_P_^2^ = 0.010). There was anecdotal evidence for a negative relationship of trait anxiety during gender decisions (BF_10_ = 1.966). However, this correlation failed the Bonferroni-corrected significance threshold. For the other correlations, moderate evidence against a relationship was found (BFs_01_ > 6; Table 2).

#### EPN

**Fig. 5:**
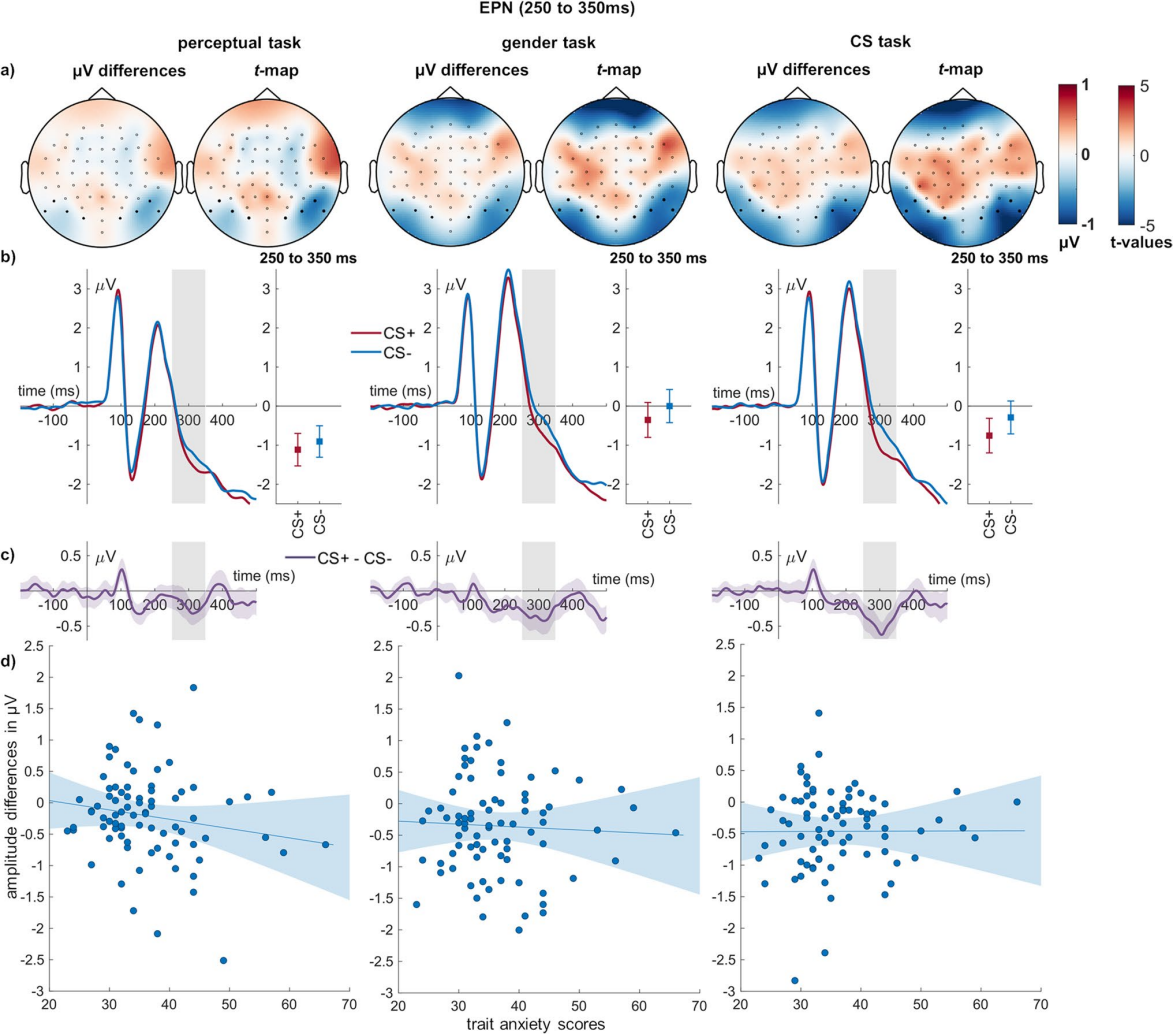
**EPN effects of conditioning and relation to individual differences. a)** In each task, scalp topographies depict the amplitude differences between CS + and CS − faces, and *t*-maps of these differences are shown. **b)** ERP waveforms show the time course over highlighted sensors. Error bars show 95% confidence intervals. **c)** Difference plots contain 95% bootstrap confidence intervals of intra-individual differences. **d)** Scatter plots of EPN differences with trait anxiety scores. The regression line and 95% confidence intervals are highlighted

For the EPN, both main effects of conditioning (*F*_(1,79)_ = 35.50, *p* < 0.001, η_P_^2^ = 0.310; Fig. 5) and task reached significance (*F*_(1.80,141.80)_ = 27.54, *p* < 0.001, η_P_^2^ = 0.259). CS + faces elicited a larger EPN than CS − faces. The perceptual task elicited a more negative EPN than the gender the CS tasks (*ps* < 0.001). The CS task also elicited a more negative EPN than the gender task (*p* < 0.001). There was a significant interaction of emotion and task on the EPN amplitude (*F*_(2,158)_ = 3.13, *p* = 0.046, η_P_^2^ = 0.038; Fig. 5). While differential effects were present in all three conditions, differences were largest in the CS task (*t*_(1,79)_ = 5.72, *p* < 0.001), followed by the gender task (*t*_(1,79)_ = 3.98, *p* < 0.001), and perceptual task (*t*_(1,79)_ = 2.67, *p* = 0.009).

Concerning relationships of EPN differences and trait anxiety, all relationships failed Bonferroni correction. There was anecdotal evidence against a relationship of trait anxiety during perceptual decisions (BF_01_ = 2.01) and moderate evidence against the remaining correlations (BFs_01_ > 5; Table 2).

#### LPP

**Fig. 6:**
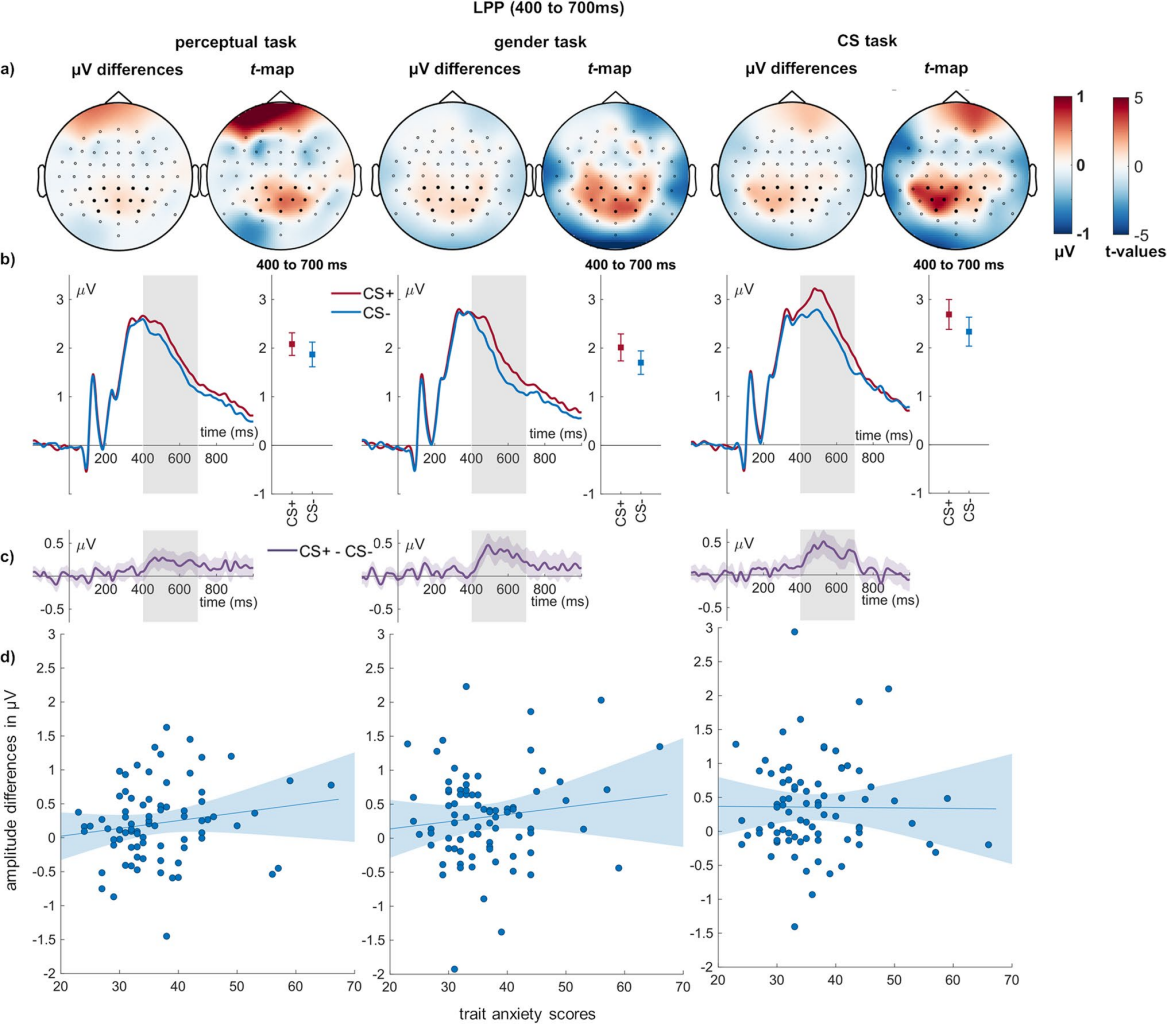
**LPP effects of conditioning and relation to individual differences. a)** In each task, scalp topographies depict the amplitude differences between CS + and CS − faces, and *t*-maps of these differences are shown. **b)** ERP waveforms show the time course over highlighted sensors. Error bars show 95% confidence intervals. **c)** Difference plots contain 95% bootstrap confidence intervals of intra-individual differences. **d)** Scatter plots of LPP differences with trait anxiety scores. The regression line and 95% confidence intervals are highlighted

For the LPP, both main effects of conditioning (*F*_(1,79)_ = 48.34, *p* < 0.001, η_P_^2^ = 0.380; Fig. 6) and task reached significance (*F*_(1.78,140.23)_ = 31.92, *p* < 0.001, η_P_^2^ = 0.288). There was no significant interaction of conditioning and task (*F*_(2,158)_ = 1.15, *p* = 0.320, η_P_^2^ = 0.014; Fig. 6). CS + faces elicited a larger LPP than CS − faces. The CS task led to larger LPP amplitudes compared with both the perceptual and the gender task (*ps* < 0.001). The latter two did not differ from one another (*p* = 0.142). Concerning relationships with trait anxiety, all relationships failed Bonferroni correction. There was again only anecdotal evidence against a relationship of trait anxiety during perceptual decisions (BF_01_ = 2.11) but moderate evidence against the remaining correlations (BFs_01_ > 3; Table 2).

## Discussion

The current study examined the impact of trait anxiety on ERP modulations to CS + versus CS − faces across three different tasks. Remarkably, we found no relationship between trait anxiety and ERP differences. Concerning task effects, we found increased amplitudes for CS + faces for early (P1, N170), mid-latency (EPN), and late (LPP) ERP components. Moreover, we observed two interactions between conditioning status and task for the P1 and EPN components: The P1 component showed the highest differentiation when participants needed to decide on perceptual information. In contrast, during the EPN, the highest differentiation between CS + and CS − faces were found when participants decided upon the CS association.

Our main goal was to derive a clear picture of how trait anxiety affects ERP responses to fear-conditioned CS + faces using a differential fear conditioning paradigm. It has been suggested that anxious individuals show increased attention to potential threat cues (MacLeod & Clarke, 2015; Mathews & Mackintosh, 1998; Mathews & MacLeod, 2002) and elevated sensitivity to detect fearful faces (Japee et al., 2009). Other studies have suggested that high trait anxiety levels are linked to deficits in disengaging threatening stimuli (Fox et al., 2001, 2002; Yiend & Mathews, 2001), or to a general lack in dealing with attentional-control processes (Derakshan & Eysenck, 2009; Eysenck et al., 2007), or to reduced discrimination of threat (Stegmann et al., 2020). ERP studies that investigated responses to negative facial expressions found inconclusive results (for the P1, see Bar-Haim et al., 2005; Holmes et al., 2008; for the N170, see Williams et al., 2007; for the EPN, see Steinweg et al., 2021; Holmes et al., 2008; Walentowska & Wronka, 2012).

The few previous ERP/ERF studies also showed inconsistent results concerning the relationship between trait anxiety and responses to fear-conditioned stimuli, showing enhanced P1/M1 and M170 responses for higher anxious individuals (Rehbein et al., 2015; You et al., 2021), no relationship between trait anxiety and LPP responses during different phases of a fear-conditioning experiment (Nelson et al., 2015; Panitz et al., 2018), or increased amplitudes of the P_D_ component with increased trait anxiety (Kappenman et al., 2021). A very recent study suggested that trait anxiety is specifically associated with increased P1 responses to visual features of CS + Gabor patches, stimulating the subcortical magnocellular pathway (You et al., 2021). Our study tested if anxiety affects ERPs to CS + versus CS − faces depending on specific task conditions. We found no effect of trait anxiety and early or late differential responses, which implies that, at least for the given range of trait anxiety scores and the used design, trait anxiety differences are not related to differential ERP responses. Remarkably, a recent study examined neutral faces associated with negative evaluative person knowledge and observed no link between trait anxiety and ERP differences (Krasowski et al., 2021). While individual differences, specifically in trait anxiety, are reasoned to relate to fear-conditioning mechanisms (Indovina et al., 2011; Lonsdorf & Merz, 2017), our study finds no support for any strong relationship between trait anxiety and responses to the CS + . Besides, our further explorations also showed no reliable relation of ERP differences with individual neuroticism and agreeableness scores (see the Supplementary Material Sect. 1; see also Brandt & Mueller, 2022). However, we cannot exclude the possibility that using extreme groups of trait anxiety scores (Rehbein et al., 2015), specific stimulus features for fear conditioning (You et al., 2021), other trait scales than trait anxiety (e.g., see suggestions by Panitz et al., 2018), and other experimental parameters of the fear-conditioning procedure might change the outcomes (see also limitations below).

Another point of view is that participants with high trait-anxiety scores might exhibit a reduced differentiation of signals associated with threat versus nonthreat (Felmingham et al., 2003; Thayer & Lane, 2000), thus an overgeneralization (Roesmann et al., 2022; Stegmann et al., 2020). This lack of discrimination could lead to a sustained condition of anxiety where all stimuli are perceived as threatening (Thayer & Lane, 2000). Remarkably, one study recently revealed that individuals scoring high in trait anxiety show increases of the P_D_ component towards threatening stimuli that are reasoned to reflect attentional suppression to eliminate the threat (Kappenman et al., 2021). Kappenman et al. (2021) interpreted their findings to show that threat elicited an initial allocation of attention regardless of the anxiety level first (to pictures and conditioned-threat cues). In contrast, higher anxiety levels were only associated with elevated suppression for conditioned threats. Such conditioned threats are suggested to exhibit more tangible signs of a real threat than pictorial threats that are less efficiently and consistently threatening, especially when anxious participants get used to them across many trials (Kappenman et al., 2021). This might explain why previous studies often showed no clear evidence of an increased attentional bias to threat among anxious individuals, depending on the type of the used stimulus (Bar-Haim et al., 2007; Kruijt et al., 2019). We can provide moderate evidence against a relationship of trait anxiety for most of the examined differential ERP modulations. However, we find only anecdotal evidence concerning EPN and LPP effects during perceptual decisions and anecdotal in favor of a relationship with N170 effects during the gender discrimination task. In our attempt to provide an overview on possible relationships with trait anxiety and tasks, and the necessity to correct for multiple comparisons, our sample size might not have been sensitive enough to detect small relationships.

Regarding the influence of the three tasks, we observed conditioning effects on all examined ERP components but task-dependent conditioning effects for the P1 and EPN components. Modulations of the P1 (Muench et al., 2016) or N170 (Camfield et al., 2016), and EPN (Ferreira de Sá et al., 2019) are less often reported compared to LPP effects (Stolz et al., 2019; Seligowski et al., 2018; Bacigalupo & Luck, 2018; Wiemer et al., 2021; Ferreira de Sá et al., 2019; but see a recent examination of the full-time range by Sperl et al., 2021). Furthermore, with a smaller subsample (*N* = 40), we observed main effects of conditioning for the N170, EPN, and LPP, but not the predicted interactions (Bruchmann et al., 2021). In contrast, our increased sample in the current study revealed P1 and EPN interactions, suggesting that these can be observed only with high statistical power. In our registration, we reasoned based on recent studies that suggested the P1 to be (at least partially) related to inhibitory processes (Klimesch et al., 2007; Lasaponara et al., 2017; Slagter et al., 2016), in contrast to the N1/N170, while both act as sensory-gain functions. Thus, this led us to predict that participants need to inhibit distracting (CS +) faces during attention to a perceptual feature, leading to larger P1 amplitudes to CS + faces during the distraction task. In contrast, the N1 relation to stimulus amplification (Eimer, 1994; Luck et al., 1990; Vogel & Luck, 2000) and task-unconstrained modulations for emotional faces (Schindler et al., 2021; Schindler et al., 2020a) led us to expect only the observed main effects of conditioning. For the EPN, we also found interactions between the task and conditioning status. These effects showed increased EPN amplitudes when deciding about the CS association. The EPN reflects early attentional selection processes based on emotional relevance (Schupp, Junghöfer, et al., 2004; Schupp, Öhman, et al., 2004) and is sensitive towards different attention tasks (Schupp et al., 2007a, 2007b; Schupp et al., 2007a, 2007b). Recent studies showed that at the level of the EPN, reliable interactions between the task and emotional modulations can be observed for faces, where decreasing attention to faces led to decreasing emotional modulation (Schindler et al., 2020a, b). During the LPP window, descriptively, the differences also when attending to the CS association, but no interaction was found. This is surprising, as the LPP is highly vulnerable to modulations or the attention focus (Hajcak et al., 2006; Rellecke et al., 2012; Schupp, et al., 2007a, 2007b; Weinberg et al., 2012). The LPP is reasoned to be generated by broad and distributed sources (Liu, Huang, et al., 2012; Liu, Keil, et al., 2012) and related to controlled attention processes (Hajcak et al., 2009; Schupp et al., 2006). We aimed to avoid any attention spillover between the task and the faces by keeping face presentation as short as possible. Given our continuous reinforcement rate (33% of the trials paired with the US), even when the CS association was task-irrelevant by instruction, one may argue that they were highly relevant from the participants’ perspective because attending to the face allowed to predict whether the US may or may not occur. Thus, participants might have processed the CS status in all tasks, possibly after completing the immediate discrimination tasks.

## Constraints on generality and future directions

With regard to our study’s results, some limitations have to be mentioned. Because the present study was limited to a laboratory setting, it is important to note that our findings might not capture real-life experiences and consequently should not be overgeneralized. We used brief presentation durations to define our results more precisely on initial stimulus processing, and we employed specific tasks to draw the participant's attention to perceptual, facial, or CS features. To avoid different learning or extinction rates across tasks and participants, we instructed participants about the CS-contingencies in advance and had a high re-enforcement rate during the tasks. Both might have caused ceiling effects. We only focused on the impact of threat-related faces based on acoustic US stimuli that were proven to be efficient in acquiring a CS + response (Camfield et al., 2016; Panitz et al., 2015; Sperl et al., 2016). Indeed, rating and pupil dilation showed successful conditioning, which was present during all three tasks (Finke et al., 2021) but unrelated to trait anxiety (Panitz et al., 2018; Torrents-Rodas et al., 2013). For future studies, it might be of particular relevance studying differential effects toward imagined US (Mueller et al., 2019) that could better capture specific relationships between trait anxiety and fear-conditioning mechanisms (Indovina et al., 2011). Furthermore, differential responses toward classic fear-conditioning might be only observed during specific phases or better predicted by trait fearfulness than trait anxiety (Panitz et al., 2018). Our study focused on trait anxiety effects during three different tasks to well-learned CS + and did not examine effects during fear acquisition, extinction, or “pure” recall. In addition, our sample contained a large number of female subjects, which might affect the generalizability of this study since previous work exhibited sex differences processing of emotional stimuli (Hampson et al., 2006; Kret & De Gelder, 2012). Our sample comprised healthy individuals with subclinical trait anxiety scores. It could be that threat-specific neural responses only differ between extreme groups in subclinical anxiety (Stegmann et al., 2020) or in clinical disorders due to pathological processes (Torrents-Rodas et al., 2013). Finally, the exploration across ERPs and tasks needed appropriate multiple comparison correction methods, while future studies might pursue the most promising relationships between trait anxiety and ERP differences between CS + and CS − faces.

## Conclusions

Our results indicate that differential processing of fear-conditioned faces in a classical conditioning paradigm is for specific information processing stages task-independent (N170, LPP), while in other time windows, these are influenced by the decision upon different aspects of the face-stimulus (P1, EPN). Most importantly, we found no correlation between ERP modulations and individual differences in trait anxiety, questioning the hypothesis that the changed information processing of conditioned threat depends on trait anxiety.

## Supplementary Information

Below is the link to the electronic supplementary material.

Supplementary file1 (DOCX 1066 KB)
