# Supplementary material for: No trait anxiety influences on early and late differential neuronal responses to aversively conditioned faces across three different tasks
Source: Cogn Affect Behav Neurosci. 2022 Mar 29;22(5):1157–71. doi: 10.3758/s13415-022-00998-x (PMC9458573; doi:10.3758/s13415-022-00998-x)
Supplement: Supplementary file 1 — Supplementary file1 (DOCX 1066 KB) [file 13415_2022_998_MOESM1_ESM.docx]

**Title: No trait anxiety influences on early and late differential neuronal responses to aversively conditioned faces across three different tasks**

**SUPPLEMENTARY MATERIALS**

Sebastian Schindler^1,2*┼^, Jana Heinemann^1┼^, , Maximilian Bruchmann^1,2^, Robert Moeck^1^, and Thomas Straube^1,2^

^1^ Institute of Medical Psychology and Systems Neuroscience, University of Muenster

^2^ Otto Creutzfeldt Center for Cognitive and Behavioral Neuroscience, University of Muenster

* Corresponding author

^┼^ shared-first authors

**Correspondence address**

Institute of Medical Psychology and Systems Neuroscience

University of Münster

Von-Esmarch-Str. 52, D-48149 Münster, Germany

e-mail: [sebastian.schindler@ukmuenster.de](mailto:sebastian.schindler@ukmuenster.de)

**1. Relationships between ERP differences and personality traits**

So far, several studies have reported how personality traits, such as neuroticism, and agreeableness lead to a differential processing of threat-related stimuli (for neuroticism, e.g., see Doty et al., 2013; for agreeableness, e.g., see Meier et al., 2006). Learning from associations seems to be further biased by personality traits, where neuroticism and agreeableness are related to learning negative associations (Vogel et al., 2019). Neuroticism and trait anxiety are highly correlated (Bishop & Forster, 2013), both reasoned to represent a preferential recognizing of faces associated with a threat (Doty et al., 2013; Mathews & Mackintosh, 1998). Finally, individuals scoring high on agreeableness seem to evaluate faces as more friendly (Czerwon et al., 2011; Knyazev et al., 2008). Neuroticism and trait anxiety (see the main document) are highly correlated (Bishop & Forster, 2013), both reasoned to represent a preferential recognizing of faces associated with a threat (Doty et al., 2013; Mathews & Mackintosh, 1998). Individuals scoring high on agreeableness seem to evaluate faces as more friendly (Czerwon et al., 2011; Knyazev et al., 2008). No electrophysiological study has yet examined such traits in a classic conditioning paradigm.

Concerning ERP findings on the impact of neuroticism and agreeableness, only a few studies only tested for personality differences in the processing of negative stimuli (see Ku et al., 2020a; Zhang et al., 2015a, 2013a; Gomez et al., 2002b; but see Speed et al., 2015b; Bartussek et al., 1996a). Those studies reported increased processing of negative content during late processing stages in high neurotic participants (Gomez et al., 2002a; Zhang et al., 2013b; Ku et al., 2020b; Zhang et al., 2015b; but see Bartussek et al., 1996b; Speed et al., 2015a). Recent studies showed that individuals high in callousness, comparable with low agreeableness, exhibit a lack of recognizing fearful faces and have reduced N170 and LPP amplitudes (Brislin & Patrick, 2019; Brislin et al., 2018). In this study, we also aimed to explore possible relationships between personality and ERP differences, since behavior studies reported associations between neuroticism and responses to faces signaling threat (see Doty et al., 2013; de Jong et al., 2009; Chan et al., 2007) and of agreeable individuals that evaluated faces as more friendly (Czerwon et al., 2011; Knyazev et al., 2008). Further, a recent study observed associations between agreeableness and EPN responses towards faces of putative criminals during a perceptual distraction task (Krasowski et al., 2021). However, we found no correlation between the two personality traits and ERP differences (see Supplementary Table S1). All correlations provide at least moderate evidence against a relationship (all BFs_01_ > 3). Most strikingly, the used distraction tasks and the association of neutral faces with negative valence closely followed a recent study showing EPN relationships during a perceptual distraction task (Krasowski et al., 2021). In contrast to this latter study, where face-identities were instructed to have committed a brutal crime (Krasowski et al., 2021), faces in this study were paired faces with a loud scream. Thus, Krasowski and colleagues (2021) study reported that the relationship might not be based on threat-processing but rather be explained by the link between agreeableness and aggressive behavior (for a meta-analysis, see Jones et al., 2011). Agreeableness relates to a sensitivity to detect anger and hostility in faces, absent during classic fear conditioning. Alternatively, the pairing of negative evaluation and neutral faces might have been viewed as conflicting information (Krasowski et al., 2021). For low agreeable participants, neutral faces paired with hostile information might have caused higher unexpectedness. Taken together, by the current absence of relationships, we concluded that agreeableness is not generally related to differential ERP processing of threat-related faces, but rather this depends on the type of the stimulus and learning of negative information.

**Supplementary Table S1: Correlation of ERP differences with neutroticism and agreeableness scores**

| P1 | correlation | perceptual task | gender task | CS task |
| --- | --- | --- | --- | --- |
|  | BF_10_ | 0.145 | 0.140 | 0.268 |
| neuroticism | Pearson's *r* | .082 | .018 | -.059 |
|  | *p*-value^a^ | .469 | .872 | .602 |
|  | BF_10_ | 0.181 | 0.141 | 0.160 |
| agreeableness | Pearson's *r* | -.083 | .044 | .001 |
|  | *p*-value^a^ | .462 | .699 | .990 |
|  | BF_10_ | 0.182 | 0.150 | 0.140 |
| N170 | correlation | perceptual task | gender task | CS task |
| neuroticism | Pearson's *r* | -.036 | -.111 | -.104 |
|  | *p*-value^a^ | .753 | .328 | .357 |
|  | BF_10_ | 0.147 | 0.223 | 0.212 |
| agreeableness | Pearson's *r* | -.106 | -.037 | -.088 |
|  | *p*-value^a^ | .348 | .742 | .440 |
|  | BF_10_ | 0.215 | 0.147 | 0.187 |
| E PN | correlation | perceptual task | gender task | emotion task |
| neuroticism | Pearson's *r* | -.032 | -.057 | -.024 |
|  | *p*-value^a^ | .776 | .613 | .831 |
|  | BF_10_ | 0.145 | 0.158 | 0.143 |
| agreeableness | Pearson's *r* | -.105 | -.159 | -.080 |
|  | *p*-value^a^ | .352 | .160 | .481 |
|  | BF_10_ | 0.214 | 0.368 | 0.178 |
| LPP | correlation | perceptual task | gender task | emotion task |
| neuroticism | Pearson's *r* | .113 | .067 | .054 |
|  | *p*-value^a^ | .317 | .556 | .636 |
|  | BF_10_ | 0.228 | 0.166 | 0.156 |
| agreeableness | Pearson's *r* | -.077 | .010 | -.024 |
|  | *p*-value^a^ | .496 | .931 | .832 |
|  | BF_10_ | 0.175 | 0.140 | 0.143 |

Note: ^a^ Bonferroni-corrected significance threshold *p* < .002. BF_10_ indicates evidence in favor of the alternative hypothesis (H1) and conversely BF_01_ evidence in favor of the null hypothesis (where BF_10_ = 1/ BF_01_).

**2. ERP identification**

We had a priori restrictions based on the common knowledge about the typical time windows and sensor regions for each ERP, which was the basis for our ERP component identification. In line with our expectation, we slightly deviated from this preregistration in time (registered N170: 120 to 170 ms; EPN 200 to 350 ms; LPP 400 to 600 ms) and space (registered P1, N170, and EPN: P9, P7, PO7, P10, P8, PO8; LPP: C1, Cz, C2, CP1, CPz, CP2). This was due to the registered approach to validate ERP windows for the P1 and N170 by collapsing ERPs across all conditions (Luck & Gaspelin, 2017). For the EPN and LPP, typically scored as differences between negative and neutral stimuli, we collapsed CS+ and CS- faces across the three attention tasks to identify differential effects. In doing so, we used visual inspection based on collapsed ERPs across all conditions for the P1 and N170 or collapsed CS+ versus CS- ERPs across all tasks for differential EPN and LPP (see Supplementary Figure S1). We thus segmented time windows in intervals from 80 to 100 ms for the P1, from 120 to 170 ms for the N170, from 250 to 350 ms for the EPN, and from 400 to 700 ms for the LPP. We measured the P1, N170, and EPN over two symmetrical occipital clusters (P1: P9, P7, PO7, P5, P10, P8, PO8, P6; N170 and EPN: P9, P7, PO7, O1; P10, P8, PO8, O2). Additionally, we measured the LPP component over a centro-parietal cluster (CP3, CP1, CPz, CP2, CP4, P3, P1, Pz, P2, P4, PO3, POz, PO4).

**
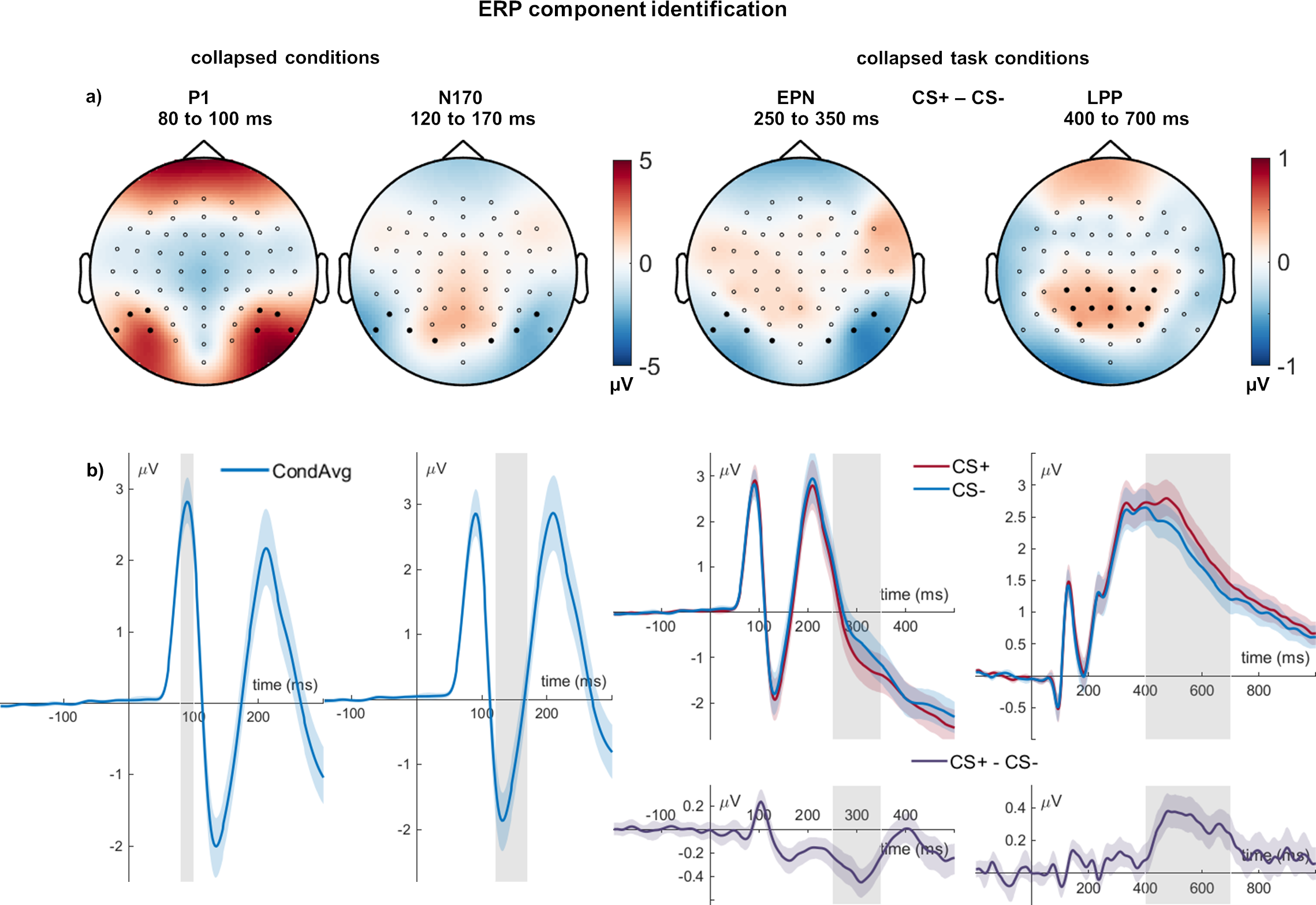
Supplementary Figure S1. ERP component identification.** **a)** Temporal evolvement. For the P1 and N170, ERPs were collapsed across all conditions. For the EPN and LPP, ERPs were collapsed ERPs across attention tasks for the difference between CS+ and CS- faces. **b)** The mean activity is displayed for the identified time windows and the identified sensors highlighted. The time course of mean ERPs and the differences between CS+ and CS- faces are displayed. For the P1 and N170, the collapsed conditions and corresponding 95% confidence intervals are displayed. For the EPN and LPP, additionally, the difference wave and corresponding 95% bootstrap confidence intervals are displayed.

**3. Exact task instructions**

Before each task, participants were given information on how to navigate through instructions first, followed by specific instructions. In italic, we provide additional information or describe additional changes on the screen.

**Translated instructions in the perceptual task:**

"In this experiment, you need two response keys *(keys highlighted).* With these two keys, you can move forward and backward through these instruction screens" *(next screen)*

"Next, you will see faces with lines are overlaid" *(Examples with horizontal and vertical lines displayed)*

"Press the left key *(key highlighted)* when these lines are horizontal. Press the right key *(key highlighted)* when these lines are vertical." *(keys counterbalanced; next screen)*

"Please constantly fixate the center of the screen (fixation cross). Avoid any head movement and try to reduce eye-blinks." *(next screen)*

"The Experiment starts when you press the Space key." *(next screen the experiment starts)*

**Gender task (first two screens and last two screens are identical):**

"Press the left key *(key highlighted)* when the face is male. Press the right key *(key highlighted)* when the face is female." *(keys counterbalanced; next screen)*

**CS task (first two screens and last two screens are identical):**

"Press the left key *(key highlighted)* when the person is sometimes followed by a quiet sound. Press the right key *(key highlighted)* when the person is sometimes followed by a loud scream." *(keys counterbalanced; next screen)*

**German original instructions in the perceptual task:**

"In diesem Experiment benötigen Sie 2 Tasten. *(Tasten hervorgehoben)* Mit diesen beiden Tasten können Sie vorwärts oder rückwärts durch die Einleitung blättern." *(nächster Bildschirm)*

"Gleich sehen Sie Gesichter, die von Linien überlagert sind." *(Beispiellinien gezeigt)*

"Drücken Sie die linke Taste *(Taste hervorgehoben)* wenn die Linien horizontal verlaufen. Drücken Sie die rechte Taste *(Taste hervorgehoben)* wenn die Linien vertikal verlaufen." *(Tasten gegenbalanciert; nächster Bildschirm)*

"Bitte schauen Sie die ganze Zeit auf die Mitte des Bildschirms (Kreuz). Vermeiden Sie jede Bewegung und versuchen Sie, möglichst wenig zu blinzeln." *(nächster Bildschirm)*

"Das Experiment startet, wenn Sie die Leertaste drücken." *(nächster Bildschirm start des Experiments)*

**Gender task (first two screens and last two screens are identical):**

"Drücken Sie die linke Taste *(Taste hervorgehoben)* wenn das Gesicht männlich ist. Drücken Sie die rechte Taste *(Taste hervorgehoben)* wenn das Gesicht weiblich ist." *(Tasten gegenbalanciert; nächster Bildschirm)*

**CS task (first two screens and last two screens are identical):**

"Drücken Sie die linke Taste *(Taste hervorgehoben)* wenn die Person ab und an von einem leisen Ton gefolgt wird. Drücken Sie die rechte Taste *(Taste hervorgehoben)* wenn die Person ab und an von einem lauten Schrei gefolgt wird." *(Tasten gegenbalanciert; nächster Bildschirm)*

**Supplementary Materials References**

Bartussek, D., Becker, G., Diedrich, O., Naumann, E., & Maier, S. (1996a). Extraversion, neuroticism, and event-related brain potentials in response to emotional stimuli. *Personality and Individual Differences*, *20*(3), 301–312. https://doi.org/10.1016/0191-8869(95)00175-1

Bartussek, D., Becker, G., Diedrich, O., Naumann, E., & Maier, S. (1996b). Extraversion, neuroticism, and event-related brain potentials in response to emotional stimuli. *Personality and Individual Differences*, *20*(3), 301–312. https://doi.org/10.1016/0191-8869(95)00175-1

Bishop, S., & Forster, S. (2013). Trait Anxiety, Neuroticism, and the Brain Basis of Vulnerability to Affective Disorder. In J. Armony & P. Vuilleumier (Eds.), *The Cambridge Handbook of Human Affective Neuroscience* (pp. 553–574). Cambridge University Press. https://doi.org/10.1017/CBO9780511843716.031

Brislin, S. J., & Patrick, C. J. (2019). Callousness and Affective Face Processing: Clarifying the Neural Basis of Behavioral-Recognition Deficits Through the Use of Brain Event-Related Potentials. *Clinical Psychological Science*, *7*(6), 1389–1402. https://doi.org/10.1177/2167702619856342

Brislin, S. J., Yancey, J. R., Perkins, E. R., Palumbo, I. M., Drislane, L. E., Salekin, R. T., Fanti, K. A., Kimonis, E. R., Frick, P. J., Blair, R. J. R., & Patrick, C. J. (2018). *Callousness and Affective Face Processing in Adults: Behavioral and Brain-Potential Indicators*. 11.

Chan, S. W. Y., Goodwin, G. M., & Harmer, C. J. (2007). Highly neurotic never-depressed students have negative biases in information processing. *Psychological Medicine*, *37*(9), 1281–1291. https://doi.org/10.1017/S0033291707000669

Czerwon, B., Lüttke, S., & Werheid, K. (2011). Age Differences in Valence Judgments of Emotional Faces: The Influence of Personality Traits and Current Mood. *Experimental Aging Research*, *37*(5), 503–515. https://doi.org/10.1080/0361073X.2011.619468

de Jong, P. J., Koster, E. H. W., van Wees, R., & Martens, S. (2009). Emotional facial expressions and the attentional blink: Attenuated blink for angry and happy faces irrespective of social anxiety. *Cognition & Emotion*, *23*(8), 1640–1652. https://doi.org/10.1080/02699930802490227

Doty, T. J., Japee, S., Ingvar, M., & Ungerleider, L. G. (2013). Fearful face detection sensitivity in healthy adults correlates with anxiety-related traits. *Emotion*, *13*(2), 183–188. https://doi.org/10.1037/a0031373

Gomez, R., Gomez, A., & Cooper, A. (2002a). Neuroticism and extraversion as predictors of negative and positive emotional information processing: Comparing Eysenck’s, Gray’s, and Newman’s theories. *European Journal of Personality*, *16*(5), 333–350. https://doi.org/10.1002/per.459

Gomez, R., Gomez, A., & Cooper, A. (2002b). Neuroticism and extraversion as predictors of negative and positive emotional information processing: Comparing Eysenck’s, Gray’s, and Newman’s theories. *European Journal of Personality*, *16*(5), 333–350. https://doi.org/10.1002/per.459

Jones, S. E., Miller, J. D., & Lynam, D. R. (2011). Personality, antisocial behavior, and aggression: A meta-analytic review. *Journal of Criminal Justice*, *39*(4), 329–337. https://doi.org/10.1016/j.jcrimjus.2011.03.004

Knyazev, G. G., Bocharov, A. V., Slobodskaya, H. R., & Ryabichenko, T. I. (2008). Personality-linked biases in perception of emotional facial expressions. *Personality and Individual Differences*, *44*(5), 1093–1104. https://doi.org/10.1016/j.paid.2007.11.001

Krasowski, C., Schindler, S., Bruchmann, M., Moeck, R., & Straube, T. (2021). Electrophysiological responses to negative evaluative person-knowledge: Effects of individual differences. *Cognitive, Affective, & Behavioral Neuroscience*. https://doi.org/10.3758/s13415-021-00894-w

Ku, L.-C., Chan, S., & Lai, V. T. (2020a). Personality Traits and Emotional Word Recognition: An ERP Study. *Cognitive, Affective, & Behavioral Neuroscience*, *20*(2), 371–386. https://doi.org/10.3758/s13415-020-00774-9

Ku, L.-C., Chan, S., & Lai, V. T. (2020b). Personality Traits and Emotional Word Recognition: An ERP Study. *Cognitive, Affective, & Behavioral Neuroscience*, *20*(2), 371–386. https://doi.org/10.3758/s13415-020-00774-9

Luck, S. J., & Gaspelin, N. (2017). How to get statistically significant effects in any ERP experiment (and why you shouldn’t). *Psychophysiology*, *54*(1), 146–157. https://doi.org/10.1111/psyp.12639

Mathews, A., & Mackintosh, B. (1998). A Cognitive Model of Selective Processing in Anxiety. *Cognitive Therapy and Research*, *22*(6), 539–560. https://doi.org/10.1023/A:1018738019346

Meier, B. P., Robinson, M. D., & Wilkowski, B. M. (2006). Turning the Other Cheek: Agreeableness and the Regulation of Aggression-Related Primes. *Psychological Science*, *17*(2), 136–142. https://doi.org/10.1111/j.1467-9280.2006.01676.x

Speed, B. C., Nelson, B. D., Perlman, G., Klein, D., N., Kotov, R., & Hajcak, G. (2015a). Personality and emotional processing: A relationship between extraversion and the Late Positive Potential in adolescence. *Psychophysiology*, *52*(8), 1039–1047. https://doi.org/10.1111/psyp.12436

Speed, B. C., Nelson, B. D., Perlman, G., Klein, D. N., Kotov, R., & Hajcak, G. (2015b). Personality and emotional processing: A relationship between extraversion and the late positive potential in adolescence: Personality and the late positive potential. *Psychophysiology*, *52*(8), 1039–1047. https://doi.org/10.1111/psyp.12436

Vogel, T., Hütter, M., & Gebauer, J. E. (2019). Is Evaluative Conditioning Moderated by Big Five Personality Traits? *Social Psychological and Personality Science*, *10*(1), 94–102. https://doi.org/10.1177/1948550617740193

Zhang, W., Zhou, R., Wang, Q., Zhao, Y., & Liu, Y. (2013a). Sensitivity of the late positive potentials evoked by emotional pictures to neuroticism during the menstrual cycle. *Neuroscience Letters*, *553*, 7–12. https://doi.org/10.1016/j.neulet.2013.06.037

Zhang, W., Zhou, R., Wang, Q., Zhao, Y., & Liu, Y. (2013b). Sensitivity of the late positive potentials evoked by emotional pictures to neuroticism during the menstrual cycle. *Neuroscience Letters*, *553*, 7–12. https://doi.org/10.1016/j.neulet.2013.06.037

Zhang, W., Zhou, R., Wang, Q., Zhao, Y., & Liu, Y. (2015a). Progesterone mediates the late positive potentials evoked by affective pictures in high neuroticism females. *Psychoneuroendocrinology*, *59*, 49–58. https://doi.org/10.1016/j.psyneuen.2015.04.023

Zhang, W., Zhou, R., Wang, Q., Zhao, Y., & Liu, Y. (2015b). Progesterone mediates the late positive potentials evoked by affective pictures in high neuroticism females. *Psychoneuroendocrinology*, *59*, 49–58. https://doi.org/10.1016/j.psyneuen.2015.04.023
